# Associations Between Apparent Diffusion Coefficient Values and Histopathologic Prognostic Factors in Breast Cancer: A Retrospective Study

**DOI:** 10.1155/ijbc/5114852

**Published:** 2026-05-05

**Authors:** Fatemeh Mahdavi Sabet, Fahimeh Zeinalkhani, Shayan Forghani, Peyman Kamali Hakim, Hadise Zeinalkhani, Fatemeh Tahanian

**Affiliations:** ^1^ Students’ Scientific Research Center, Tehran University of Medical Sciences, Tehran, Iran, tums.ac.ir; ^2^ Advanced Diagnostic and Interventional Radiology Research Center (ADIR), Tehran University of Medical Sciences, Tehran, Iran, tums.ac.ir; ^3^ School of Medicine, Tehran University of Medical Sciences, Tehran, Iran, tums.ac.ir; ^4^ Department of Radiology, Iran University of Medical Sciences, Tehran, Iran, iums.ac.ir; ^5^ Department of Radiology, Shahid Beheshti University of Medical Sciences, Tehran, Iran, sbmu.ac.ir

**Keywords:** apparent diffusion coefficient (ADC), breast cancer, diffusion-weighted imaging (DWI), magnetic resonance imaging (MRI), prognostic factors

## Abstract

**Background:**

This study is aimed at investigating the correlation between apparent diffusion coefficient (ADC) values obtained from diffusion‐weighted imaging (DWI) and prognostic factors in breast cancer. We hypothesized that lower ADC values would be observed in more aggressive tumors, including those with higher histological grades and Ki‐67 expression levels. A retrospective analysis was conducted on patients with malignant breast lesions who underwent breast magnetic resonance imaging (MRI), including DWI sequences, at our center between January 2022 and January 2023. MRI was performed on a 1.5 T scanner using a bilateral phased‐array eight‐channel breast coil. ADC values were calculated utilizing *b* values of 400 and 800 s/mm^2^ and evaluated in relation to prognostic factors, including age, tumor size, tumor grade, lymph node involvement, Ki‐67 proliferation index, estrogen receptor (ER), progesterone receptor (PR), and human epidermal growth factor receptor 2 (HER2) status.

**Results:**

This study included 88 patients with 89 malignant lesions. ADC values showed no significant association with age, tumor size, histological type, lymph node involvement, or receptor status (ER, PR, and HER2). However, tumors with higher Ki‐67 expression exhibited significantly lower median ADC values. For Ki‐67 thresholds of ≥ 14% and ≥ 30%, *p* values were 0.02 and < 0.01, respectively. A negative correlation between ADC values and the Ki‐67 index was noted (*p* = 0.01, *ρ* = −0.28). Additionally, Grade I tumors had higher ADC values than Grade II and III tumors (*p* < 0.01), while no difference was observed between Grade II and III tumors (*p* = 0.61).

**Conclusion:**

Lower ADC values correlate with higher tumor grades and Ki‐67 expression, suggesting their potential role as imaging biomarkers for assessing breast cancer aggressiveness.

## 1. Background

Breast cancer (BC) comprises a diverse and heterogeneous group of diseases, presenting with a varied spectrum of morphologies, treatment responses, and prognoses [1–3]. Prognostic markers, such as hormone receptor (HR) expression, human epidermal growth factor receptor 2 (HER2) status, Ki‐67 expression, histological grade, tumor size, and axillary lymph node involvement, are widely used to determine prognosis and guide disease management [1, 2, 4]. Typically, molecular subtype classification and treatment decisions rely on tissue biopsy, which is prone to selection bias [5]. Precise preoperative evaluation and histological assessment of prognosis are crucial to yield optimal management [6–8], and further refinement of these evaluations may enhance overall survival and clinical outcomes for patients [9, 10]. In this regard, noninvasive imaging modalities offer valuable insights and can help minimize the need for biopsies [11].

Various noninvasive modalities, including mammography, ultrasonography, and MRI, are utilized to evaluate BC diagnosis and preoperative staging [9, 12]. MRI is commonly used to determine the lesions’ morphology and contrast enhancement patterns, providing information on tumor biology and the number and extent of the lesions [3, 4, 7]. This technique outperforms mammography and ultrasonography with higher sensitivity, although its specificity remains limited [13]. Diffusion‐weighted imaging (DWI) has emerged as a noncontrast functional MRI technique for detecting breast malignancies and has recently been integrated into multiparametric MRI along with dynamic contrast‐enhanced MRI (DCE‐MRI) [14]. DWI and its quantitative parameter, the apparent diffusion coefficient (ADC), utilize the Brownian motion of water molecules to provide insight into the tumor cellularity and its microstructural characteristics [9, 15]. Several studies have demonstrated an inverse correlation between ADC values and tumor cellularity [16]. Several other factors, such as perfusion due to tumor angiogenesis, membrane integrity, and fluid viscosity, can also alter ADC values [17].

DWI and the derived ADC parameter have been thoroughly investigated for their ability to distinguish malignancy from benign lesions [18, 19]. Malignant tumors exhibit lower ADC values than benign or normal breast tissues, reflecting restricted water diffusion in these lesions [6]. ADC values have also been explored as biomarkers for assessing response to neoadjuvant therapy [20–22]. Despite these advancements, the association of ADC values with BC prognostic factors remains to be fully elucidated with inconclusive and controversial results among previously executed studies. Establishing clear associations can enhance MRI’s role as a noninvasive tool for detection, risk stratification, and personalized treatment planning. Consequently, in this study, we aimed to investigate the potential correlation between ADC values and BC prognostic factors such as Ki‐67 proliferation index, tumor size, tumor grade, ER, PR, and HER2 status.

## 2. Methods

This study aimed to evaluate the correlation between ADC values derived from DWI and key prognostic factors in patients with BC, including tumor size, grade, Ki‐67 proliferation index, ER, PR, and HER2 status. We conducted a retrospective cross‐sectional study at a tertiary academic hospital, analyzing breast MRI scans performed between January 1, 2022, and January 31, 2023.

### 2.1. Patients

This retrospective study reviewed all breast MRI examinations performed at our center during the specified period. The study was approved by our Institutional Review Board and Ethics Committee (IR.TUMS.IKHC.REC.1403.23), which granted a waiver of informed consent.

Female patients aged ≥ 18 years with biopsy‐proven BC who underwent MRI including DWI sequences and were scheduled for surgical treatment were eligible. Malignancy was confirmed by histopathological analysis of surgical specimens. Patients were excluded if they had received neoadjuvant chemotherapy or endocrine therapy, had undergone excisional biopsy before MRI, had low or nondiagnostic DWI image quality, lacked pathological confirmation, or did not proceed to surgery. Lesions smaller than 1 cm, including DCIS or invasive carcinomas with an invasive component smaller than 1 cm, and complex masses with only a minimal solid portion were also excluded because reliable ADC measurement was not feasible.

Ultimately, 88 patients with 89 breast lesions were included in the final analysis, as one patient had two lesions.

### 2.2. MRI Acquisition and Analysis

MRI examinations were performed in a standard prone position using a 1.5 T MRI scanner (General Electric Medical Systems, United States) equipped with a bilateral phased‐array eight‐channel breast coil. The imaging protocol included an axial T1‐weighted sequence without fat suppression, an axial T2‐weighted fast spin echo with fat saturation, and axial STIR sequences with and without water suppression. In‐phase and out‐of‐phase gradient‐echo sequences, as well as fat‐only and water‐only images using LAVA‐Flex, were also obtained. For dynamic contrast‐enhanced imaging, a 3D fat‐suppressed spoiled gradient‐echo sequence was acquired in five postcontrast phases following intravenous injection of 0.2 mmol/kg gadolinium‐DTPA (Dotarem, Guerbet), followed by a 15 mL saline flush. Subtraction images were generated automatically from the pre‐ and postcontrast series, along with maximum intensity projection (MIP) reconstructions in coronal and sagittal planes. Finally, a diffusion‐weighted echo‐planar imaging sequence with spatial fat suppression was acquired 10 min postcontrast using b‐values of 400 and 800 s/mm^2^. ADC maps were automatically generated using the manufacturer’s software.

Two radiologists (F.Z. with over 10 years of experience and F.T. with 4 years of experience) independently reviewed the MR images. Final interpretations were reached through consensus, with any disagreements resolved through mutual discussion. The region of interest (ROI) was manually outlined on the area of greatest diffusion restriction within the solid component of mass lesions, or the most conspicuous area of nonmass enhancement, as seen on the ADC map. ROIs were chosen based on regions with clearly high signal intensity on DWI. Efforts were made to exclude cystic, necrotic, fatty regions, and hematomas within the mass, and in cases of multifocal or multicentric lesions, the ROI was placed on the largest malignant lesion. Calculations were performed using the mean ADC values obtained from the ROI.

### 2.3. Histological Analysis

The final diagnosis was confirmed through histological examination of tissue samples obtained via surgical excision. The pathology report was documented for each lesion, including histological type classified according to the WHO guidelines [23], and tumor grade was assessed using the Nottingham modification of the Bloom–Richardson grading system [24]. This method evaluates nuclear pleomorphism, mitotic count, and tubule formation, each scored from 1 to 3. The total score classifies tumors as Grade 1 (scores of 3–5), Grade 2 (scores of 6 or 7), and Grade 3 (scores of 8 or 9). HR status (ER, PR, and HER2) and Ki‐67 proliferation index were also documented. HER2 status was determined using immunohistochemical (IHC) staining, and when equivocal, it was confirmed by fluorescence in situ hybridization (FISH). HER2 was deemed negative for IHC scores of 0 or 1+, equivocal for 2+, and positive for 3+. For cases with a 2+ IHC score, HER2 positivity was confirmed if FISH testing showed HER2 gene amplification. The Ki‐67 proliferation index was classified as positive for staining levels of 14% or higher and negative for levels below 14% to facilitate comparison with previous studies. Additionally, following the St. Gallen/Vienna 2021 guidelines, Ki‐67 positivity was also defined as ≥ 30% for a more nuanced categorization [25]. The ER and PR status was considered positive if the expression was 10% or more. Patients were further categorized as follows: Luminal A (ER or PR positive and HER2 negative), Luminal B (ER or PR positive and HER2 positive), HER2‐enriched (ER and PR negative and HER2 positive), and triple‐negative (ER, PR, and HER2 negative), following established criteria [26]. Tumor size was considered the longest diameter of the tumor.

### 2.4. Statistical Analysis

Statistical analysis was performed using the R statistical environment v. 4.3.2 (R Core Team, 2023). Descriptive statistical methods were applied to analyze the study data, including mean, standard deviation, median, frequency, ratio, and minimum and maximum values. The normality of quantitative data distribution was assessed using the Shapiro–Wilk test. For comparing normally distributed quantitative data between two groups, an independent *t*‐test was employed, while the Mann–Whitney test was applied for data not conforming to normal distribution. The Kruskal–Wallis test was utilized for comparisons among three or more groups with non‐normally distributed data, followed by the Bonferroni–Dunn test for pairwise analyses. On the other hand, the ANOVA test was utilized to compare three or more groups conforming to a normal distribution. The Spearman’s correlation test was used to investigate the correlation between ADC values and tumor size, tumor grade, age, Ki‐67 value, ER value, and PR value. Receiver operating characteristic (ROC) curve analysis was conducted, and the area under the curve (AUC) with a 95% confidence interval was determined to establish the best cutoff ADC value in differentiating distinct IHC and pathological features of BC. Additionally, diagnostic metrics such as sensitivity, specificity, positive predictive value (PPV), and negative predictive value (NPV), along with ROC curve analysis, were employed to establish parameter cutoff values. The 2‐tailed *p* value was considered statistically significant if *p* < 0.05.

## 3. Results

A total of 88 female patients involving 89 breast lesions were included in this study, with an age range from 25 to 73 years (median age 45). The most frequent histological type was invasive ductal carcinoma (IDC), accounting for 74 lesions (83%). Table 1 provides a summary of the patients’ demographic and histopathological characteristics, along with the statistical analysis of median ADC values across various prognostic subgroups. The ADC values ranged from 0.47 to 2.55 × 10^−3^ mm^2^/s, with a median of 1.04 × 10^−3^ mm^2^/s.

**Table 1:** Association of clinicopathological characteristics with ADC tumor values.

Variable	No. (%)	ADC tumor value (median, IQR) (× 10^−3^ mm^2^/s)	*p* value
Age of diagnosis (years)			0.948
≤ 50	62 (70.46%)	1.030 (0.891, 1.247)	
> 50	26 (29.54%)	1.041 (0.902, 1.184)	
Tumor histological subtype			
IDC	74 (83.15%)	1.007 (0.895, 1.214)	0.272
ILC	11 (12.36%)	1.046 (0.861, 1.133)	
DCIS	4 (4.49%)	1.463 (1.232, 1.621)	
Tumor histological grade			
1	17 (19.10%)	1.218 (1.024, 1.372)	**0.012**
2	51 (57.30%)	0.981 (0.879, 1.167)	
3	21 (23.60%)	0.995 (0.860, 1.105)	
Tumor size (mm)			
< 20 mm	30 (33.71%)	1.079 (0.928, 1.173)	0.766
20–50 mm	47 (52.81%)	0.966 (0.895, 1.155)	
> 50 mm	12 (13.48%)	1.005 (0.806, 1.325)	
Molecular subtype			
Luminal A	53 (59.55%)	1.014 (0.891, 1.176)	0.479
Luminal B (HER2 positive)	17 (19.10%)	0.958 (0.861, 1.182)	
Luminal B (HER2 negative)	0 (0.0%)	—	
Triple negative	12 (13.48%)	1.000 (0.829, 1.240)	
HER2 enriched	7 (7.87%)	1.175 (1.121, 1.288)	
Lymph node involvement			
Node positive	54 (60.67%)	0.999 (0.902, 1.157)	0.528
Node negative	35 (39.33%)	1.086 (0.833, 1.296)	
ER status			
Positive	71 (79.78%)	1.003 (0.891, 1.185)	0.484
Negative	18 (20.22%)	1.124 (0.883, 1.277)	
PR status			
Positive	60 (67.42%)	1.003 (0.887, 1.176)	0.242
Negative	29 (32.58%)	1.094 (0.916, 1.266)	
HER2 status			
Positive	24 (26.97%)	1.082 (0.901, 1.263)	0.640
Negative	65 (73.03%)	1.000 (0.888, 1.181)	
Ki‐67 expression index			
≥ 14%	63 (70.79%)	0.980 (0.867, 1.173)	**0.021**
< 14%	26 (29.21%)	1.109 (0.967, 1.368)	
≥ 30%	35 (39.33%)	0.907 (0.825, 1.087)	**p** < 0.01
< 30%	54 (60.67%)	1.090 (0.916,1.316)	

*Note:* The association between clinicopathological characteristics and ADC tumor values in the study population. The median ADC values (with interquartile ranges) are presented for different subgroups defined by age, tumor histological subtype and grade, tumor size, molecular subtype, lymph node involvement, hormone receptor status, HER2 status, and Ki‐67 expression index. Bold values indicate statistically significant results (*p* < 0.05).

Abbreviations: ADC, apparent diffusion coefficient; DCIS, ductal carcinoma in situ; ER, estrogen receptor; HER2, human epidermal growth factor receptor 2; IDC, invasive ductal carcinoma; ILC, invasive lobular carcinoma; IQR, interquartile range; MRI, magnetic resonance imaging; PR, progesterone receptor.

Comparison of ADC values between DCIS and invasive carcinomas (IDC and ILC combined) revealed no statistically significant difference (*p* = 0.12). Similarly, no significant difference was observed in ADC values between IDC and ILC tumors (*p* = 0.65).

No significant correlation was observed between the ADC values and patients’ age (*p* = 0.76, *ρ* = −0.03) or between ADC values and age subgroups (*p* = 0.95). The lesion sizes ranged from 6 to 90 mm, with a median of 24 mm. There was no significant difference between median ADC values and tumor size groups (*p* = 0.77), and the correlation analysis was also insignificant (*p* = 0.61, *ρ* = −0.06).

**Figure 1:**
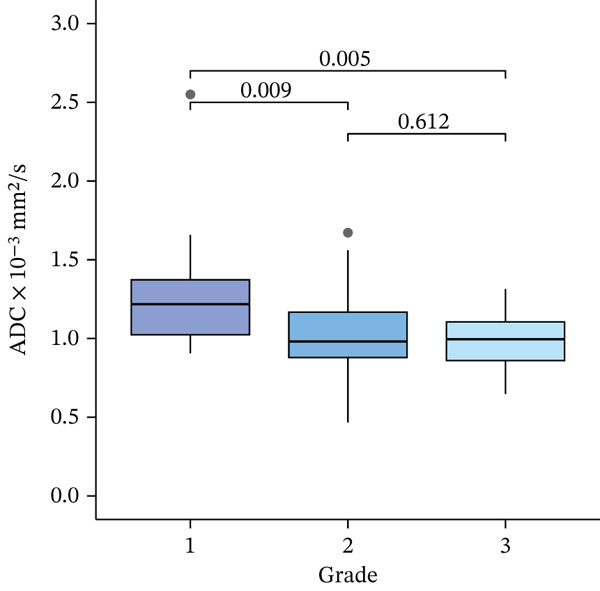
Boxplot illustrating the relationship between ADC values (× 10^−3^ mm^2^/s) and histological grade subgroups of breast carcinomas (Grade I, Grade II, and Grade III).

There was a significant correlation between histological grade subgroups and ADC values (*p* = 0.01). The median ADC values for Grade II and Grade III tumors were significantly lower than those for Grade I tumors, with a *p* value of < 0.01 for both comparisons. However, there was no significant difference in ADC values between Grade II and III tumors (*p* = 0.61) (Figure 1).

**Figure 2:**
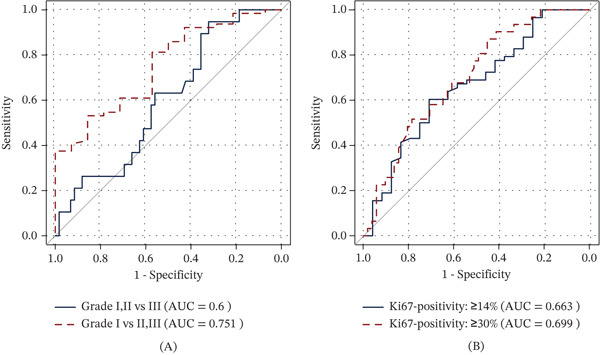
(A) Receiver operating characteristic (ROC) curves of apparent diffusion coefficient (ADC) values for differentiating Grade I tumors from Grade II and III tumors and for differentiating Grade I and II tumors from Grade III tumors. (B) ROC curve of ADC values for identifying Ki‐67 positive tumors at ≥ 14% and ≥ 30% thresholds.

In addition, we performed ROC analysis to determine the ADC value thresholds. ROC curve analysis revealed a cutoff value of 0.998 × 10^−3^ mm^2^/s to differentiate Grade I from Grade II and III tumors with a sensitivity, specificity, PPV, NPV, and accuracy of 53.1%, 85.7%, 94.4%, 28.6%, and 59.0%, respectively. The AUC was 0.75 (95% CI: 0.61–0.88). The threshold for differentiation between Grades I and II from Grade III tumors was 1.177 × 10^−3^ mm^2^/s with a sensitivity, specificity, PPV, NPV, and accuracy of 94.7%, 32.2%, 31.0%, 95.0%, and 47.4%, respectively. The AUC was 0.60 (95% CI: 0.46–0.74) (Figure 2A).

The majority of the lesions were ER positive (77.5%), PR positive (65.2%), and HER2 negative (68.5%). Lymph node involvement was observed in most cases (60.7%). No significant differences in median ADC values were found across various prognostic subgroups, including histological type, lymph node involvement, molecular subtypes, and ER, PR, or HER2 status. Regarding Ki‐67 expression, a positivity threshold of ≥ 14% classified 61 patients (68.5%) as positive and 24 patients (27.0%) as negative. When the positivity threshold was set at ≥ 30%, 52 patients (58.4%) were deemed positive, and 33 patients (37.1%) were negative. Lesions with positive Ki‐67 expression showed significantly lower ADC values, with a *p*‐value of 0.02 for the ≥ 14% cutoff and < 0.01 for the ≥ 30% cutoff.

**Figure 3:**
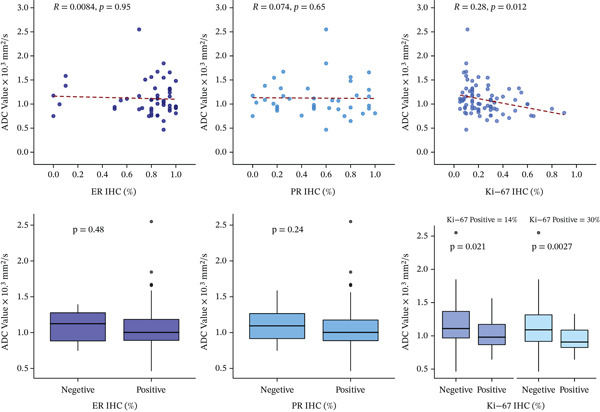
Scatterplots and boxplots illustrating the relationship between ADC values and immunohistochemical markers: (a) estrogen receptor (ER) expression, (b) progesterone receptor (PR) expression, and (c) Ki‐67 proliferation index.

Additionally, we performed ROC curve analysis to determine the ADC values threshold for predicting Ki‐67 status. For the ≥ 14% threshold, the analysis identified an ADC value of 1.04 × 10^−3^ mm^2^/s, differentiating Ki‐67‐positive tumors from Ki‐67‐negative ones with a sensitivity of 61.4%, specificity of 72.0%, PPV of 83.3%, NPV of 45.0%, and an overall accuracy of 64.6%. The AUC was 0.66 (95% CI: 0.53–0.80). For the ≥ 30% threshold, the ROC analysis revealed an ADC value threshold of 1.16 × 10^−3^ mm^2^/s, achieving a sensitivity of 87.1%, specificity of 45.1%, PPV of 49.1%, NPV of 85.2%, and an accuracy of 61.0%. The AUC was 0.70 (95% CI: 0.58–0.81) (Figure 2B). Also, an inverse correlation between Ki‐67 values and ADC values (*p* = 0.01, *ρ* = −0.28) was further found. We did not find a significant correlation between ADC values and estrogen or progesterone expression on IHC (Figure 3).

On MRI, 70 lesions (78.7%) were classified as masses, while 19 (21.3%) were identified as nonmass enhancements. The ADC values of mass lesions were significantly lower than those of nonmass enhancements, measuring 0.99 × 10^−3^ and 1.32 × 10^−3^ mm^2^/s, respectively (*p* < 0.01).

Tumor grade differed significantly between the two groups, with mass lesions demonstrating higher tumor grades than NME lesions (*p* = 0.03). In contrast, Ki‐67 did not differ significantly between the two lesion types, either when analyzed as a continuous variable (*p* = 0.37) or when categorized using a positivity threshold of ≥ 30% (*p* = 0.15).

**Figure 4:**
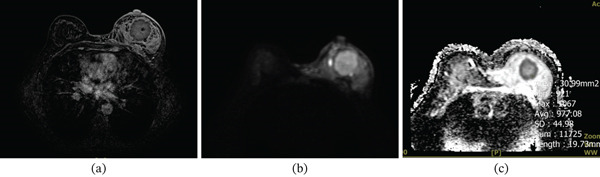
(a) Axial contrast‐enhanced T1 subtraction, (b) diffusion‐weighted imaging (DWI), and (c) ADC map sequences of breast MRI. The images show a large, heterogeneous, irregular enhancing mass with adjacent nonmass enhancement in the left breast suggesting a malignant lesion. On DWI, the mass demonstrates restricted diffusion, with an ADC value of 0.977 × 10^−3^ mm^2^/s. The final diagnosis is invasive ductal carcinoma (IDC) with a Ki‐67 proliferation index of 30% and tumor histologic Grade 2.

**Figure 5:**
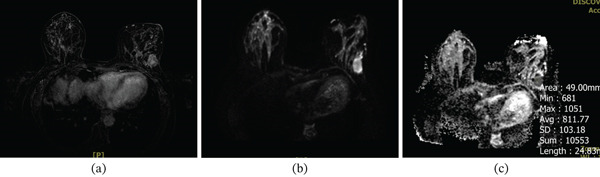
(a) Axial contrast‐enhanced T1 subtraction, (b) diffusion‐weighted imaging (DWI), and (c) ADC map sequences of breast MRI. The images show a heterogeneous, irregular enhancing mass in the left breast suggesting a malignant lesion. On DWI, the mass demonstrates restricted diffusion, with an ADC value of 0.811 × 10^−3^ mm^2^/s. The final diagnosis is invasive ductal carcinoma (IDC) with a Ki‐67 proliferation index of 45% and tumor histologic Grade 3.

**Figure 6:**
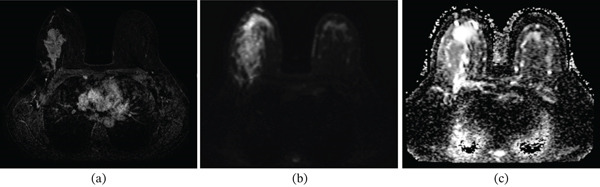
(a) Axial contrast‐enhanced T1 subtraction, (b) diffusion‐weighted imaging (DWI), and (c) ADC map sequences of breast MRI. The images show a heterogeneous, irregular enhancing mass in the right breast suggesting a malignant lesion. On DWI, the mass demonstrates restricted diffusion, with an ADC value of 1.32 × 10^−3^ mm^2^/s. The final diagnosis is invasive ductal carcinoma (IDC) with a Ki‐67 proliferation index of 10% and tumor histologic Grade 1.

Representative breast lesions are shown in Figures 4, 5, and 6.

## 4. Discussion

DWI has emerged as a valuable technique for assessing the microstructural features and changes in tumors, leading to improved diagnostic accuracy and specificity [27]. The ADC, derived from DWI, quantifies the average displacement of water molecules per unit of time [1], offering insights into tissue cellularity and membrane permeability [12]. Recent studies highlight the benefits of integrating DWI and the derived ADC for differentiating benign and malignant tumors [18, 19]. However, the role of DWI in predicting prognostic factors remains controversial. The present study investigated the association between ADC values and BC prognostic factors in BC patients treated in our center.

The median ADC value of 1.04 × 10^−3^ mm^2^/s observed in our study is compatible with previously reported values, including those by Belli et al. (1.02 × 10^−3^ mm^2^/s) [28], Costantini et al. (1.04 × 10^−3^ mm^2^/s) [8], Martincich et al. (1.07 × 10^−3^ mm^2^/s) [3], and Cipolla et al. (1.09 × 10^−3^ mm^2^/s) [29].

In concordance with the literature [2, 30], we found significantly lower ADC values in mass lesions compared with nonmass enhancements. This difference can be attributed to the indistinct boundaries in nonmass enhancements, resulting in the inadvertent inclusion of normal parenchymal tissue within the ROI [30]. In addition, mass lesions in our cohort showed significantly higher tumor grades compared with nonmass enhancements, which may also contribute to lower ADC values due to higher tumor cellularity.

The restricted diffusion of water molecules in the densely packed cells of IDC tissue results in lower ADC values [31]. Previous studies by Choi et al. and Surov et al. have reported significantly higher ADC values in DCIS than in IDC [31, 32]. Furthermore, Costantini et al. found higher ADC values in IDC tumors compared to ILC [8]. In this study, the difference in ADC values was not statistically significant despite the higher ADC values observed in DCIS lesions. This aligns with some of the previous research [3, 4]. Our study did not replicate these findings, possibly due to the limited number of ILC and DCIS cases in our patient population.

Tumor size is a critical prognostic factor, as larger tumors are linked to lymph node positivity and lower survival rates [17]. However, in alignment with most previous studies [12, 17, 27, 28], our study found no significant correlation between tumor size and ADC values. In contrast, Razek et al. reported a significant association between tumor size and ADC values, with larger tumors exhibiting lower ADC values [33].

Tumor grade is of high importance in BC evaluation, as it provides key insights into tumor behavior and long‐term prognosis [34]. Grading is determined by the degree of tumor cell differentiation and represents cellularity and tumor aggressiveness [2]. Accordingly, we hypothesized that higher grade tumors would exhibit lower ADC values. In alignment with those previously reported [8, 9, 28, 33], we found an inverse correlation between ADC values and tumor grade (*p* = 0.01, *ρ* = −0.29). However, several studies have reported no significant differences in ADC values across tumor grades [3, 31, 35, 36].

We used ROC analysis to determine a cutoff ADC value of 0.998 × 10^−3^ mm^2^/s to differentiate Grade I from Grade II and III tumors. This cutoff is higher than those reported in other studies, such as Rupa et al. (0.82 × 10^−3^ mm^2^/s) [34] and Azzam et al. (0.93 × 10^−3^ mm^2^/s) [7]. The discrepancy in cutoff values may reflect differences in sample histology, as our study included a variety of invasive cancer types, whereas Azzam et al. focused exclusively on IDCs, and Rupa et al. excluded DCIS lesions entirely [7, 34]. Variability in MRI devices and DWI acquisition parameters could also contribute to these differences. Interestingly, the cutoff reported by Surov et al. (1.03 × 10^−3^ mm^2^/s) [32] is the closest to ours, possibly due to a similar distribution of histological types in our patient groups. Additionally, Rupa et al. found that minimum ADC values offered greater specificity in distinguishing Grade I from higher grades [34]; as our study analyzed mean ADC values, we cannot make this comparison. We also established a cutoff value of 1.177 × 10^−3^ mm^2^/s for distinguishing Grade I and II tumors from Grade III tumors. The lower cutoff reported by Azzam et al. (0.79 × 10^−3^ mm^2^/s) [7], can be attributed to the aforementioned reasons.

Ki‐67 is a nuclear antigen expressed during the proliferation phase of the cell cycle, serving as an indicator of cellular proliferative activity [2, 6, 36]. This marker is commonly used to assess patients’ prognosis, pathological complete response, overall and disease‐free survival rates, and likelihood of metastasis [31, 32]. Higher Ki‐67 expression typically reflects greater cellular proliferation, which may correspond with lower ADC values, as observed in our study. We observed a weak inverse correlation between Ki‐67 expression and ADC values (*ρ* = −0.28), consistent with findings from Aydin et al., who reported a similar correlation (*ρ* = −0.27) [6]. However, Hegazy and Azzam reported a stronger inverse correlation (*ρ* = −0.78) [37].

Comparison across studies is challenging, as previous research has employed various Ki‐67 expression thresholds to differentiate tumors with low and high proliferative activity. While a 14% threshold for Ki‐67 positivity has been commonly applied in earlier research [3, 9, 12, 29], the St. Gallen/Vienna 2021 guidelines recommend a higher threshold of 30%, as it better identifies patients who may benefit from chemotherapy [25]. Therefore, we analyzed our patients by utilizing both thresholds. We found significantly lower ADC values in Ki‐67‐positive tumors than in Ki‐67‐negative tumors, applying both thresholds. Notably, the 30% threshold showed a stronger association, with a lower *p* value (< 0.01) compared to the 14% threshold (*p* = 0.02). While some studies, such as those by De Felice et al., Martincich et al., and Aydin et al., found no significant differences in ADC between Ki‐67‐positive and negative groups [1, 3, 6], others, in alignment with our results, have demonstrated a significant correlation between Ki‐67 positivity and lower ADC values [2, 12]. Luo et al. have further demonstrated that changes in ADC tumor values can reflect changes in Ki‐67 expression following neoadjuvant chemotherapy [38].

Furthermore, we established optimal ADC cutoff values of 1.04 × 10^−3^ and 1.16 × 10^−3^ mm^2^/s to differentiate Ki‐67‐positive from Ki‐67‐negative tumors based on the 14% and 30% thresholds, respectively. Tanişman et al. reported an ADC cutoff value of 1.07 × 10^−3^ mm^2^/s for distinguishing Ki‐67‐positive and negative tumors; however, their study defined Ki‐67 positivity using a 20% proliferation index threshold [2]. These findings underscore the variability in ADC cutoff values, which is largely influenced by differing Ki‐67 thresholds used across studies.

Axillary lymph node involvement is a crucial prognostic factor, significantly affecting survival outcomes and guiding treatment strategies [4]. Since this parameter denotes the advanced stage of the disease [33], we hypothesized lower ADC values in LN‐positive tumors. Most of the previously executed studies have reported accelerated ADC values in LN‐negative tumors compared to LN‐positive tumors [4, 9, 12, 28, 33]. A study by Kamitani et al. conversely demonstrated higher ADC values in LN‐positive tumors. They attributed their result to micronecroses or fibrosis within the lymph node–positive tumors [39]. Despite the lower values in LN‐positive tumors in our study, this difference remained insignificant, in alignment with some studies [17, 36]. Moutinho‐Guilherme et al. attributed their findings to the relatively smaller lesions in their study population. A similar rationale applies to our study, as most lesions were < 50 mm due to the exclusion of patients who had received neoadjuvant or endocrine therapy [17].

HER2 overexpression induces cell growth, contributing to carcinogenesis [9]. Consequently, HER2‐positive tumors are typically more aggressive, with higher cell proliferation and a greater propensity for metastasis [31]. Therefore, lower ADC values are expected in HER2‐positive tumors. However, Ren et al. conversely demonstrated higher ADC values in HER2‐positive tumors. They attributed their result to HER2‐driven angiogenesis, which enhances tumor perfusion and may offset the expected reduction in ADC caused by restricted diffusion [9]. Similarly, our study observed higher ADC values in HER2‐positive tumors, although the difference remained statistically insignificant.

Higher cellularity has been reported in HR‐positive tumors compared to HR negative [40]. Moreover, ER and PR inhibit angiogenesis, resulting in decreased perfusion and, thus, lower ADC values [39]. Therefore, we hypothesized lower ADC values in HR‐positive tumors compared to HR negative. Consistent with some of those previously reported, our study did not demonstrate a significant correlation between ADC values and ER [4, 12], PR [17, 27, 35, 36], and molecular subtypes [12]. A recent meta‐analysis by Meyer et al. demonstrated that ADC values cannot reliably distinguish between BC subtypes, concluding that ADC cannot be used to predict tumor receptor status [41]. Our findings are consistent with these conclusions; however, we recognize that the relatively small sample size, especially within certain molecular subtypes, may have limited our ability to detect significant differences. Notably, literature results regarding the correlation between ER, PR, and HER2 status and ADC values remain inconclusive and vary between different populations. Hence, we recommend that future research be conducted on bigger population samples with a particular emphasis on specific BC subtypes.

The inconsistency in the literature can be attributed to several factors, such as the choice of ROI placement and the presence of nonmass enhancements in the study population, since the diagnostic accuracy of ADC is limited for these lesions [41]. Different *b*‐values may influence ADC measurements, as higher *b*‐values require longer TE, which can increase image distortion and reduce signal‐to‐noise ratio. Furthermore, very low *b*‐values increase sensitivity to perfusion effects, which may artificially elevate ADC measurements [36]. The European Society of Breast Imaging (EUSOBI) consensus guideline on breast DWI recommends the use of at least two *b*‐values for standardized ADC calculation, typically including a low *b*‐value close to 0–50 s/mm^2^ and a high *b*‐value of approximately 800 s/mm^2^ [42]. In the present study, ADC values were calculated using *b*‐values of 400 and 800 s/mm^2^. Although the lowest *b*‐value differs from the near‐zero value recommended for standardization, the use of a higher baseline *b*‐value reduces the contribution of perfusion‐related signal components that are prominent at very low *b*‐values [21, 43, 44]. Malignant breast lesions frequently exhibit increased angiogenesis and microvascular density, and intravoxel incoherent motion (IVIM) associated with microperfusion can artificially elevate ADC measurements at low *b*‐values [45]. By using a baseline *b*‐value of 400 s/mm^2^, the influence of microperfusion is partially suppressed, allowing the calculated ADC to more closely reflect tissue diffusion properties related to tumor cellularity. The EUSOBI consensus also recognizes that DWI acquisition parameters may vary between institutions depending on scanner capabilities and clinical protocols [42]. Nevertheless, this methodological difference should be considered when directly comparing absolute ADC values from this cohort with those reported in studies using a baseline *b*‐value near 0 s/mm^2^ or other *b*‐value combinations.

Whether scanner field strength significantly affects ADC values remains controversial. Moutinho‐Guillherme et al. found no significant difference between ADC values obtained from 1.5 and 3.0 T devices [17], whereas Jeh et al. reported significantly lower ADC values obtained from 1.5 T scanners [35].

The present study has several limitations. Its retrospective design and single‐center setting may limit the generalizability of the findings. Additionally, the inclusion of nonmass lesions increases the possibility of nonparenchymal tissues being involved in the ROI. Another limitation of our study is the use of a 1.5 T MRI scanner with an eight‐channel breast coil. While this setup is still commonly used in clinical practice, it does not reflect the most advanced imaging technology currently available. This technical factor may have influenced the sensitivity of our protocol in detecting subtle differences between tumor subtypes. Furthermore, our study had a relatively small sample size, which may have influenced the lack of significance in certain correlations.

## 5. Conclusions

ADC values obtained from DWI correlate with Ki‐67 positivity and higher histological grades of breast carcinomas. In this context, we conclude that ADC values can be beneficial for assessing the biological aggressiveness of BC.

**Nomenclature**
ADC: apparent diffusion coefficient
DWI: diffusion‐weighted imaging
MRI: magnetic resonance imaging
Ki‐67: Ki‐67 proliferation index
ER: estrogen receptor
PR: progesterone receptor
HR: hormone receptor
LN: lymph node
HER2: human epidermal growth factor receptor 2
BC: breast cancer
DCE‐MRI: dynamic contrast‐enhanced magnetic resonance imaging
IDC: invasive ductal carcinoma
DCIS: ductal carcinoma in situ
ILC: invasive lobular carcinoma
IHC: immunohistochemical (staining)
FISH: fluorescence in situ hybridization
ROI: region of interest
TR: repetition time
TE: echo time
BW: bandwidth
FOV: field of view
NEX: number of excitations
MIP: maximum intensity projection
ROC: receiver operating characteristic
AUC: area under the curve
PPV: positive predictive value
NPV: negative predictive value

## Funding

No funding was received for this manuscript.

## Ethics Statement

This study was performed in accordance with the Declaration of Helsinki and was approved by our Institutional Review Board and Ethics Committee (IR.TUMS.IKHC.REC.1403.239) which granted a waiver of consent.

## Consent

The authors have nothing to report.

## Conflicts of Interest

The authors declare no conflicts of interest.

## Data Availability

The data supporting this study’s findings are available from the authors, but restrictions apply to their availability. These data were used under ethical and institutional regulations from Imam Khomeini Hospital for the current study and are therefore not publicly available. Data may, however, be made available from the authors upon reasonable request and with the necessary permissions from relevant ethics committees.
